# The use of the SF-36 questionnaire in adult survivors of childhood cancer: evaluation of data quality, score reliability, and scaling assumptions

**DOI:** 10.1186/1477-7525-4-77

**Published:** 2006-10-05

**Authors:** Raoul C Reulen, Maurice P Zeegers, Crispin Jenkinson, Emma R Lancashire, David L Winter, Meriel E Jenney, Mike M Hawkins

**Affiliations:** 1 Centre for Childhood Cancer Survivor Studies, University of Birmingham, Department of Public Health and Epidemiology, Edgbaston, Birmingham, B15 2TT, UK; 2 Unit of Genetic Epidemiology, Department of Public Health and Epidemiology, Birmingham, University of Birmingham, UK; 3 Department of General Practice, Comprehensive Cancer Institute Limburg, Catholic University of Leuven, Leuven, Belgium; 4 Health Services Research Unit, Department of Public Health, University of Oxford, Institute of Health Sciences, Oxford, UK; 5 Children's Hospital for Wales, Paediatric Oncology Department, Heath Park, Cardiff, UK

## Abstract

**Background:**

The SF-36 has been used in a number of previous studies that have investigated the health status of childhood cancer survivors, but it never has been evaluated regarding data quality, scaling assumptions, and reliability in this population. As health status among childhood cancer survivors is being increasingly investigated, it is important that the measurement instruments are reliable, validated and appropriate for use in this population. The aim of this paper was to determine whether the SF-36 questionnaire is a valid and reliable instrument in assessing self-perceived health status of adult survivors of childhood cancer.

**Methods:**

We examined the SF-36 to see how it performed with respect to (1) data completeness, (2) distribution of the scale scores, (3) item-internal consistency, (4) item-discriminant validity, (5) internal consistency, and (6) scaling assumptions. For this investigation we used SF-36 data from a population-based study of 10,189 adult survivors of childhood cancer.

**Results:**

Overall, missing values ranged per item from 0.5 to 2.9 percent. Ceiling effects were found to be highest in the role limitation-physical (76.7%) and role limitation-emotional (76.5%) scales. All correlations between items and their hypothesised scales exceeded the suggested standard of 0.40 for satisfactory item-consistency. Across all scales, the Cronbach's alpha coefficient of reliability was found to be higher than the suggested value of 0.70.

Consistent across all cancer groups, the physical health related scale scores correlated strongly with the Physical Component Summary (PCS) scale scores and weakly with the Mental Component Summary (MCS) scale scores. Also, the mental health and role limitation-emotional scales correlated strongly with the MCS scale score and weakly with the PCS scale score. Moderate to strong correlations with both summary scores were found for the general health perception, energy/vitality, and social functioning scales.

**Conclusion:**

The findings presented in this paper provide support for the validity and reliability of the SF-36 when used in long-term survivors of childhood cancer. These findings should encourage other researchers and health care practitioners to use the SF-36 when assessing health status in this population, although it should be recognised that ceiling effects can occur.

## Background

Dramatic improvements in anti-cancer therapy over the last few decades have resulted in a growing number of long-term survivors of childhood cancer. Although childhood cancer has become an increasingly more curable disease, the effects of this disease and its treatment may have profound long-term effects on the health of survivors. Due to this potential for adverse late effects, the health status of survivors of childhood cancer has been more studied over recent years. However, the number of studies focussing on *self-perceived *health of survivors is fairly limited, so that more research in this area is warranted.

An instrument that is designed to measure self-perceived health status is the SF-36 health survey questionnaire. This questionnaire has been used in a number of previous studies that have investigated the health status of childhood cancer survivors [1-7], but it never has been evaluated regarding data quality, scaling assumptions, and reliability in this population.

As health status among childhood cancer survivors is being increasingly investigated, it is important that the measurement instruments are reliable, validated and appropriate for use in this population. Although the psychometric criteria of the SF-36 have been demonstrated previously in the general population and in other patient groups, it is unknown whether these criteria are also applicable to the group of survivors of childhood cancer and should therefore be tested empirically [8,9].

The aim of this study was to assess the data quality, score reliability, and scaling assumptions of the SF-36 questionnaire in more than 10,000 long-term survivors of childhood cancer. This evaluation was based on the largest population-based study of adult survivors of childhood cancer to date.

## Methods

### Data collection

This study used data from the British Childhood Cancer Survivor Study (BCCSS), which is a population-based cohort study of survivors of childhood cancer who were 16 years or older at the time of recruitment. The cohort included all individuals who had been diagnosed with childhood cancer between 1940 and 1991, in Britain, and who had survived for at least 5 years. From 2000 to 2005, 14,450 survivors were mailed a questionnaire ascertaining issues related to adverse health outcomes. For those who did not mail the questionnaire back to the Study Centre, up to three postal reminders were sent in the following weeks. If a questionnaire was ultimately not returned then this was considered as an implicit refusal. The questionnaire contained the standard form of the SF-36 (version 1) which was administered at the beginning of the full questionnaire, as recommended by its developers [10].

Data on cancer diagnosis were obtained from the National Registry of Childhood Tumours. Written informed consent was obtained from each participant, and the study was approved by the Multi-Centre Research Ethics Committee and each of the 212 Local Research Ethics Committees.

### SF-36

The SF-36 is a generic health questionnaire, which contains 36 items that measure eight dimensions (scales) of health status. The eight dimensions are: physical functioning (PF), role limitation-physical (RP), role limitation-emotional (RE), social functioning (SF), mental health (MH), energy and vitality (EV), bodily pain (BP), and general health perception (GH). Scores on each scale range from 0–100, with a score of 100 indicating the highest rating of health. In addition, a Mental Component Summary scale (MCS) and a Physical Component Summary (PCS) scale can be derived from these eight scales by factor analysis. Because of the large sample size no imputation algorithms were used to obtain scores for missing values.

### Method of analysis

To assess whether the SF-36 is an appropriate tool for measuring health status among survivors of childhood cancer we used the following recommended criteria [11,12]: (1) data completeness, (2) distribution of the scale scores, (3) item-internal consistency, (4) item-discriminant validity, (5) internal consistency reliability, and (6) scaling assumptions of the PCS and MCS. These criteria were evaluated for the whole group of survivors, for survivors with specific types of cancer, for survivors of different age groups, for both male and female survivors, and for survivors with different ages at diagnosis. These stratifications were done in order to take possible heterogeneity due to these factors into account.

First, the number of completed items and the amount of missing data in every item were determined in order to assess *data quality*. Second, the *distribution of the scale scores *was evaluated by assessing the percentage of lowest (floor) and highest (ceiling) scores on the different scales. Third, *item-internal consistency *was evaluated by calculating the correlation of every item with its hypothesised scale (corrected for overlap). A correlation of above 0.40 has been suggested as being supportive of item-internal consistency [10]. Fourth, *item-discriminant validity *was examined by comparing the correlation between an item and its hypothesised scale versus the correlation of that same item with a supposedly unrelated scale. This comparison was performed for every item and every scale. A difference of more than two standard errors between the values of the correlations was accepted as a statistically significant difference. Scaling success rates were calculated as the proportion of successful comparisons relative to the total number of comparisons. A comparison was deemed successful whenever an item correlated significantly higher with its hypothesised scale than with another, unrelated, scale [10]. Fifth, Chronbach's alpha coefficient of reliability was used to evaluate the *internal consistency *of the SF-36. A Cronbach's alpha coefficient of 0.70 or greater was considered satisfactory [12]. Lastly, to evaluate the *scaling assumptions of the PCS and MCS*, both the scale scores were derived by means of confirmatory factor analysis and correlations of every SF-36 scale with the PCS and MCS scales were calculated. In order to support the two-factor structure of the PCS and MCS, the physical health related scales (PF, RP, BP) should correlate strongly (r ≥ 0.7) with the PCS and weakly (r ≤ 0.3) with the MCS. Similarly, the mental health related scales (MH, RE) should correlate strongly (r ≥ 0.7) with the MCS and weakly (r ≤ 0.3) with the PCS. The EV and GHP scales should correlate moderately to strongly (≥ 0.3) with both summary scales, and the SF scale should correlate strongly (r ≥ 0.7) with the MCS and moderately (0.3 < r < 0.7) with the PCS.

## Results

Seventy percent (n = 10,189) of the survivors returned the questionnaire and completed at least one item on the SF-36. Eighty-eight percent (n = 8,934) of those survivors completed all items on the questionnaire, so that all scales could be calculated. Missing values ranged per item from 0.5 to 2.9 percent, with items on the role limitation-emotional scale having the highest percentage of missing values (range: 2.6–2.9%). There was no increase in missing value rates in items near the end of the questionnaire.

For the survivor group as a whole, individual SF-36 scales could not be calculated for only a small percentage of survivors (range: 1.8–3.9%). However, this percentage was slightly higher among survivors of CNS tumours (range: 3.3–6.5%). The percentage of missing values in each scale did not depend on sex, age at questionnaire completion, or age at diagnosis (results not shown).

Overall, floor effects were most pronounced in the role-limitation physical and role-limitation emotional scales, but were relatively small (4.2%, 9.9% respectively). Ceiling effects were found to be highest in the role limitation-physical (76.7%) and role limitation-emotional (76.5%) scales. Ceiling effects decreased with increase in age at questionnaire completion, particularly in the physical health related scales (Table 1).

**Table 1:** Percentage of survivors scoring highest score possible (ceiling).

	PF	RP	BP	GH	EV	SF	RE	MH
	
all survivors	49.9	76.7	52.3	9.9	3.5	58.9	76.5	3.7
**Cancer Diagnosis**								
leukaemia	56.3	82.6	57.0	12.0	5.0	63.6	79.9	4.5
Hodgkin's disease	57.7	80.9	53.9	5.5	2.6	61.3	77.7	2.4
non-Hodgkin's	56.6	81.3	55.3	10.4	5.0	59.4	75.1	3.3
CNS	34.2	63.3	49.1	7.6	2.1	46.7	69.9	2.8
neuroblastoma	45.9	77.3	50.1	9.8	4.2	58.8	77.3	4.4
retinoblastoma	63.0	80.3	56.0	14.9	3.6	65.8	77.6	4.6
Wilms' tumour	55.4	81.8	52.4	9.4	2.8	63.8	79.4	3.8
bone tumour	18.1	61.9	27.2	6.7	2.1	50.1	74.5	4.1
soft tissue sarcoma	51.2	77.4	49.5	10.9	3.8	60.1	75.4	4.4
other	55.8	81.9	54.7	10.0	3.2	64.5	79.2	3.6
								
**Sex**								
Males	53.1	77.5	56.0	11.0	5.0	62.4	77.7	5.1
Females	42.5	71.3	45.6	8.1	1.7	52.3	70.1	2.1
								
Age*								
16–19	56.8	82.4	58.5	12.8	6.3	64.3	79.0	5.3
20–29	54.4	78.9	55.7	10.6	3.8	58.8	77.1	3.9
30–39	49.1	75.8	50.7	8.9	2.3	57.7	76.0	3.2
40–49	39.9	69.9	45.0	7.5	2.2	56.8	73.0	2.5
50+	21.6	63.2	34.7	6.7	1.9	53.6	74.8	2.7
								
**Age at diagnosis**								
0–3	51.4	76.6	53.5	12.0	4.2	60.0	75.5	4.3
4–7	50.2	75.8	53.2	9.5	3.6	58.6	74.3	4.2
8–11	44.2	72.2	49.4	7.7	2.6	53.5	73.0	2.9
12–16	41.8	70.8	46.6	7.1	2.3	54.9	71.5	2.4

*Age at questionnaire completion

PF: Physical Functioning; RP: Role-Limitation Physical; BP; Bodily Pain;

GH: General Health Perception; EV: Energy/Vitality; SF: Social Functioning;

RE: Role-Limitation Emotional: MH: Mental Health

Table 2 shows the range of item-scale correlations for all survivors and by cancer diagnosis, sex, age at questionnaire completion, and age at diagnosis. All correlations between items and their hypothesised scales exceeded the suggested standard of 0.40 for satisfactory item-consistency. Overall, the correlations between items and scales other than their hypothesised scale all were lower than correlations between items and their hypothesised scale. However, item 9d (How much time during the last month have you felt calm and peaceful?) correlated slightly higher with the energy/vitality scale than with its hypothesised scale (MH) among the overall cohort of survivors, survivors of Wilms' tumours, Hodgkin's disease, and non-Hodgkin's lymphomas, females, survivors younger than 39, and those diagnosed before the age of three.

**Table 2:** Item-internal consistency reliability coefficients and scaling failures (indicated by one or more stars).

	PF	RP	BP	GH	EV	SF	RE	MH
	
all survivors	0.69–0.88	0.75–0.82	0.81	0.61–0.81	0.71–0.74	0.67	0.70–0.76	0.52–0.73*
**Cancer diagnosis**								
leukaemia	0.64–0.84	0.74–0.80	0.82	0.63–0.80	0.69–0.71	0.59**	0.67–0.74	0.47–0.72
Hodgkin's disease	0.62–0.84	0.72–0.83	0.79	0.51–0.78	0.73–0.75	0.74	0.70–0.74	0.58–0.75*
non-Hodgkin's	0.640.84	0.73–0.83	0.81	0.61–0.81	0.75–0.77	0.67	0.69–0.75	0.53–0.73*
CNS	0.70–0.89	0.75–0.82	0.82	0.59–0.81	0.69–0.73	0.67	0.73–0.79	0.48–0.69
neuroblastoma	0.64–0.90	0.79–0.83	0.82	0.58–0.84	0.70–0.74	0.62	0.66–0.75	0.51–0.76
retinoblastoma	0.65–0.89	0.69–0.81	0.79	0.55–0.78	0.70–0.73	0.61	0.67–0.73	0.54–0.76
Wilms' tumour	0.63–0.84	0.72–0.81	0.80	0.61–0.82	0.70–0.75	0.68	0.69–0.77	0.54–0.75*
bone tumour	0.48–0.86	0.75–0.84	0.82	0.49–0.81	0.71–0.76	0.71	0.80–0.85	0.56–0.76
soft tissue sarcoma	0.56–0.86	0.70–0.80	0.80	0.56–0.82	0.73–0.77	0.70	0.68–0.78	0.58–0.80
other	0.62–0.84	0.78–0.84	0.79	0.59–0.82	0.72–0.76	0.69	0.68–0.74	0.54–0.73
								
**Sex**								
Males	0.68–0.88	0.73–0.81	0.79	0.57–0.80	0.71–0.74	0.66	0.70–0.75	0.51–0.72
Females	0.68–0.88	0.76–0.83	0.83	0.58–0.82	0.70–0.73	0.67	0.71–0.77	0.51–0.73*
								
**Age**^†^								
16–19	0.66–0.88	0.66–0.74	0.77	0.58–0.80	0.66–0.71	0.57**	0.69–0.65	0.48–0.70*
20–29	0.68–0.87	0.73–0.81	0.80	0.59–0.81	0.71–0.75	0.65	0.69–0.76	0.53–0.74*
30–39	0.68–0.87	0.76–0.84	0.83	0.56–0.81	0.72–0.75	0.69	0.72–0.77	0.53–0.74*
40–49	0.71–0.90	0.80–0.85	0.81	0.59–0.83	0.74–0.76	0.73	0.72–0.81	0.55–0.74
50+	0.62–0.88	0.76–0.87	0.83	0.55–0.81	0.66–0.74	0.69	0.78–0.82	0.49–0.71
								
**Age at diagnosis**								
0–3	0.69–0.88	0.74–0.82	0.80	0.58–0.81	0.71–0.72	0.64**	0.69–0.76	0.50–0.72*
4–7	0.68–0.88	0.72–0.80	0.82	0.57–0.80	0.69–0.75	0.63**	0.69–0.75	0.52–0.72
8–11	0.68–0.88	0.76–0.83	0.82	0.58–0.83	0.73–0.75	0.70	0.73–0.77	0.52–0.74
12–16	0.64–0.88	0.78–0.84	0.81	0.56–0.81	0.72–0.75	0.71	0.71–0.78	0.55–0.74

*Scaling failure:item 9d correlated higher with EV than with MH **Scaling failure: item 6 correlated higher with RE than with SF

^†^Age at questionnaire completion

PF: Physical Functioning; RP: Role-Limitation Physical; BP; Bodily Pain; GH: General Health Perception; EV: Energy/Vitality;

SF: Social Functioning; RE: Role-Limitation Emotional: MH: Mental Health

Also, among survivors of leukaemia, those younger than 19, and those diagnosed before the age of 7, item 6 (To what extent have your physical or emotional problems interfered with your normal social activities?) correlated higher with the role-emotional scale than with its hypothesised scale (SF). The number of scaling failures did not exceed two in any group, giving a scaling success rate of at least 99.3% indicating high item-discriminant validity.

Across all scales, the Cronbach's alpha coefficient of reliability was found to be higher than the suggested value of 0.70, with values ranging from 0.73 to 0.96 across the different cancer groups (Table 3).

**Table 3:** Internal consistency reliability coefficients (Cronbach's alpha).

	PF	RP	BP	GH	EV	SF	RE	MH
	
all survivors	0.95	0.91	0.87	0.87	0.87	0.79	0.86	0.84
**Cancer Diagnosis**								
leukaemia	0.94	0.90	0.87	0.87	0.86	0.73	0.84	0.82
Hodgkin's disease	0.93	0.90	0.84	0.84	0.88	0.85	0.85	0.86
non-Hodgkin's	0.94	0.90	0.86	0.87	0.89	0.80	0.84	0.85
CNS	0.96	0.91	0.88	0.88	0.86	0.78	0.88	0.92
neuroblastoma	0.95	0.91	0.88	0.87	0.87	0.76	0.83	0.85
retinoblastoma	0.95	0.89	0.85	0.85	0.87	0.75	0.84	0.86
Wilms' tumour	0.93	0.90	0.86	0.87	0.87	0.81	0.86	0.85
bone tumour	0.93	0.91	0.89	0.85	0.88	0.83	0.91	0.85
soft tissue sarcoma	0.94	0.89	0.86	0.87	0.88	0.82	0.86	0.86
other	0.94	0.92	0.84	0.87	0.88	0.81	0.83	0.85
								
**Sex**								
Males	0.95	0.90	0.85	0.86	0.87	0.78	0.85	0.83
Females	0.95	0.91	0.88	0.88	0.87	0.79	0.86	0.84
								
Age*								
16–19	0.95	0.86	0.82	0.85	0.85	0.72	0.82	0.81
20–29	0.95	0.90	0.85	0.87	0.87	0.78	0.85	0.84
30–39	0.95	0.91	0.88	0.87	0.88	0.81	0.86	0.85
40–49	0.96	0.93	0.88	0.88	0.88	0.82	0.88	0.84
50+	0.95	0.93	0.89	0.87	0.86	0.80	0.90	0.82
								
**Age at diagnosis**								
0–3	0.95	0.90	0.86	0.87	0.87	0.77	0.85	0.83
4–7	0.95	0.90	0.87	0.87	0.86	0.76	0.85	0.84
8–11	0.95	0.91	0.87	0.87	0.88	0.81	0.87	0.84
12–16	0.95	0.92	0.87	0.87	0.88	0.82	0.87	0.85

*Age at questionnaire completion

PF: Physical Functioning; RP: Role-Limitation Physical; BP; Bodily Pain;

GH: General Health Perception; EV: Energy/Vitality; SF: Social Functioning;

RE: Role-Limitation Emotional: MH: Mental Health

Overall, the derived PCS and MCS scales together explained 70.9% of the total variance. Consistent across all cancer groups, the physical health related scale scores (PF, RP, and BP) correlated strongly with the PCS scale scores and weakly with the MCS scale (Table 4). Also, MH and RE correlated strongly with the MCS scale score and weakly with the PCS scale score. Moderate to strong correlations with both summary scores were found for the general health perception, energy/vitality, and social functioning scales. These findings were consistent with correlations that have been obtained in previous studies involving the general population in the US [13] and the UK [14]. However, there was a small discrepancy between survivors and UK norm population data in the correlation between the RE and the PCS scale, this correlation being slightly higher among childhood cancer survivors. Also, among survivors of age 16 to 19, the BP scale showed a correlation below 0.7 (r = 0.55) with the PCS scale and a correlation above 0.3 with the MCS scale (r = 0.49). These violations were not found or at least much less pronounced among older survivors.

**Table 4:** Correlation between scale scores and PCS/MCS scale scores.

	PCS								MCS							
		
	PF	RP	BP	GH	EV	SF	RE	MH	PF	RP	BP	GH	EV	SF	RE	MH
		
UK norms [14]	0.79	0.75	0.78	0.64	0.38	0.51	0.11	0.13	0.05	0.26	0.20	0.42	0.73	0.65	0.78	0.89
**Cancer Diagnosis**																
all survivors	0.85	0.82	0.73	0.60	0.40	0.62	0.27	0.13	0.13	0.27	0.32	0.53	0.76	0.59	0.73	0.91
leukaemia	0.83	0.81	0.69	0.55	0.33	0.63	0.28	0.13	0.14	0.24	0.36	0.59	0.80	0.57	0.68	0.90
Hodgkin's disease	0.83	0.81	0.72	0.55	0.34	0.61	0.20	0.16	0.09	0.27	0.37	0.55	0.78	0.60	0.77	0.89
non-Hodgkin's	0.85	0.77	0.78	0.70	0.40	0.54	0.25	0.13	0.13	0.29	0.25	0.40	0.78	0.65	0.79	0.92
CNS	0.88	0.82	0.67	0.66	0.47	0.67	0.32	0.11	0.10	0.28	0.37	0.48	0.71	0.54	0.70	0.91
neuroblastoma	0.81	0.80	0.71	0.50	0.32	0.55	0.20	0.07	0.04	0.31	0.45	0.62	0.78	0.67	0.75	0.88
retinoblastoma	0.83	0.83	0.75	0.54	0.31	0.50	0.28	0.05	0.16	0.21	0.26	0.55	0.80	0.64	0.75	0.92
Wilms' tumour	0.85	0.81	0.77	0.67	0.42	0.58	0.24	0.18	0.15	0.30	0.30	0.40	0.75	0.66	0.78	0.90
bone tumour	0.82	0.84	0.85	0.54	0.39	0.60	0.25	0.11	0.23	0.22	0.24	0.54	0.77	0.61	0.77	0.92
soft tissue sarcoma	0.85	0.80	0.78	0.67	0.44	0.52	0.19	0.14	0.11	0.27	0.28	0.44	0.70	0.68	0.81	0.90
other	0.83	0.77	0.80	0.61	0.45	0.60	0.25	0.16	0.17	0.34	0.24	0.52	0.73	0.61	0.78	0.90
																
**Sex**																
Males	0.86	0.81	0.71	0.56	0.35	0.61	0.27	0.11	0.12	0.28	0.35	0.57	0.77	0.59	0.71	0.91
Females	0.85	0.82	0.75	0.63	0.43	0.63	0.27	0.12	0.14	0.27	0.29	0.49	0.75	0.59	0.75	0.91
																
Age*																
16–19	0.84	0.82	0.55	0.47	0.28	0.62	0.27	0.10	0.11	0.25	0.49	0.62	0.82	0.58	0.67	0.89
20–29	0.86	0.80	0.71	0.59	0.40	0.59	0.22	0.14	0.10	0.28	0.30	0.52	0.75	0.61	0.76	0.91
30–39	0.85	0.81	0.75	0.57	0.36	0.62	0.27	0.13	0.15	0.28	0.30	0.54	0.78	0.60	0.73	0.90
40–49	0.85	0.84	0.76	0.69	0.44	0.68	0.37	0.14	0.19	0.26	0.31	0.46	0.74	0.56	0.70	0.92
50+	0.84	0.82	0.82	0.66	0.55	0.65	0.26	0.19	0.23	0.28	0.22	0.50	0.65	0.56	0.75	0.90
																
**Age at diagnosis**																
0–3	0.85	0.82	0.71	0.59	0.36	0.62	0.31	0.11	0.11	0.28	0.36	0.53	0.79	0.59	0.71	0.91
4–7	0.86	0.82	0.69	0.60	0.41	0.65	0.24	0.13	0.11	0.24	0.35	0.53	0.74	0.57	0.74	0.90
8–11	0.87	0.80	0.75	0.59	0.40	0.58	0.27	0.12	0.13	0.31	0.30	0.53	0.75	0.61	0.74	0.91
12–16	0.84	0.82	0.79	0.59	0.40	0.61	0.24	0.13	0.18	0.26	0.26	0.54	0.76	0.61	0.76	0.91

*Age at questionnaire completion

PF: Physical Functioning; RP: Role-Limitation Physical; BP; Bodily Pain; GH: General Health Perception; EV: Energy/Vitality; SF: Social Functioning;

RE: Role-Limitation Emotional: MH: Mental Health

## Discussion

The findings of the present study suggest that the SF-36 exhibits good validity and reliability when used in long-term survivors of childhood cancer. Apart from ceiling effects observed in some scales, most properties were satisfactory with regards to conventional psychometric criteria.

Eighty-eight percent of the 10,189 subjects completed all items, which is identical to the 88% observed in the UK general population [14,15]. The percentage of missing values per item ranged between 0.5 to 2.9 percent, indicating that no particular item on the SF-36 had a substantially higher completion rate than other items. Missing values per item were roughly similar to those found in a UK normative general population sample (range: 0.43–1.91) [14,15].

Ceiling effects were found to be highest in the role limitation-physical (79.0%) and role limitation-emotional (77.3%) scales. The ceiling effect observed in these two scales can be explained, at least partly, by the dichotomous format (yes vs. no) of the items (4a-d, 5a-c) that measure these concepts. Ceiling effects in the role limitation-physical and role limitation-emotional scale scores have been found in other populations [10], so that this effect cannot not solely be attributed to the specific responses in childhood cancer survivors. The limitations of these dichotomous items have been recognised by their developers and, in the newer version of the SF-36 (version 2), a 5-point Likert scale has been used, in stead of the dichotomous scale. According to the developers, this should reduce the ceiling and floor effects generally observed within these items and, thereby, should improve precision.

The ceiling effects observed in the PF, RP, BP, SF, and RE scale scores may be a consequence of the relative youth of the childhood cancer survivor cohort. A young population generally has better health status than the general population, consequently, it will tend to score higher on most scales, resulting in more common ceiling effects. When comparing these ceiling effects between cancer survivors and a sample from the general population [10,14] with a similar age distribution, comparable percentages of ceiling effects were found (results not shown), indicating that the ceiling effects we observed are not specifically related to the population of childhood cancer survivors. The observed ceiling effects in these scales may however be a result of the lack of sensitivity of these scales in general. However, in large studies (n > 100) these ceiling effects should not cause large difficulties when testing whether two groups differ statistically from each other with regard to their mean score on a SF-36 scale. It has been shown that, probably as a result of the Central Limit Theorem, the use of parametric methods, such as a t-test, when comparing means are fairly robust against violations of non-normality [16]. The question whether the use of a mean is an appropriate measure in the presence of non-normality however remains. It is therefore advisable, in addition to reporting mean scores or mean differences, to report scale scores and scale score differences at the median and outer centiles such as for example the 25^th ^and 75^th ^[14,17]. This will give the potential reader insight in the nature of the actual spread of the scale scores.

Item-internal consistency was acceptable, as all item-scale correlations exhibited a value that exceeded 0.40. According to our findings, there is substantial evidence of discriminant validity of the SF-36 scales in adult survivors of childhood cancer. This implies that the SF-36 scales can discriminate between the different concepts that should be measured.

Cronbach's alpha exceeded 0.70 for all scales, indicating high internal consistency. The reliability, therefore, is high. However, the high alpha coefficients observed may be partly due to the ceiling effects observed in some scales.

The PCS and MCS scales explained over 70% of the total variance, which is somewhat higher than the 66% of variance explained by these scales in the UK normative data [14,15]. Obtained correlations between the eight scales and the PCS and MCS scales in our data largely were similar to the correlations between these eight scales and the PCS and MCS scales found in the UK normative data [14,15]. Although survivors showed a slightly higher correlation between the role-emotional scale and the PCS than the general population, this might be attributed to the fact that the prevalence of physical problems associated with role emotional problems is higher among childhood cancer survivors than in the general population. For example, neurological problems are more common among childhood cancer survivors, and may affect the survivor both physically and mentally. The moderate correlation of the bodily pain scale with both the PCS and MCS scales among young survivors (16–19 years) suggests that those survivors who experience bodily pain are likely to be more mentally than physically affected by bodily pain than survivors of older age. Nonetheless, the overall findings support the scaling assumptions of the SF-36 scales in our data, indicating that a two-factor model can be used in evaluating health status among long-term survivors of childhood cancer.

Because of the population-based design of our study, the validity and reliability of the SF-36 was assessed in a representative sample of all childhood cancer survivors in the UK. Respondents were similar to non-respondents with respect to age, sex, diagnosis of childhood cancer, and cancer treatment (Reulen *et al*. Health status of adult survivors of childhood cancer: a large scale population-based study from the British Childhood Cancer Survivor Study (submitted)).

A limitation of this study was that only cross-sectional data was available and we were therefore not able to assess the responsiveness of the SF-36 over time. However, as survivors within this study will be followed-up over time, and probably will complete another SF-36 questionnaire, we probably will have the opportunity to assess the responsiveness in future analyses. Hence, the findings in this paper are only applicable to the use of the SF-36 in studies comparing childhood cancer survivors to other comparison groups.

In conclusion, the findings presented in this paper provide support for the validity and reliability of the SF-36 when used in long-term survivors of childhood cancer. These findings should encourage other researchers and health care practitioners to use the SF-36 when assessing health status in this population, bearing in mind, however, its susceptibility to ceiling effects. The ceiling effects observed were however not specific to the group of childhood cancer survivors and may therefore indicate the lack of sensitivity of some SF-36 scales in general.

## Competing interests

The author(s) declare that they have no competing interests.

## Authors' contributions

MMH designed the study, and contributed to the analysis and interpretation of data, and to the preparation of the manuscript. RCR conducted the statistical analysis, interpreted the data, and drafted the manuscript. MPZ contributed to the interpretation of the data and critical revision of the manuscript. CJ contributed to the conception, interpretation of the data, and critical revision of the manuscript. ERL, DWL and MEJ contributed to the conception of the study and critical revision of the manuscript. All authors have read and approved the final version of this manuscript.
